# Are Scientists Sufficiently Ambitious?

**DOI:** 10.1093/function/zqac048

**Published:** 2022-09-08

**Authors:** Ole H Petersen

**Affiliations:** School of Biosciences, Cardiff University, Sir Martin Evans Building, Cardiff CF10 3AX, UK

Some years ago, I went to an art exhibition in Barcelona that posed an interesting question to other artists as well as the viewing public. Are contemporary artists sufficiently ambitious? The conclusion of those organizing the exhibition was negative. The kind of ambition they were concerned with was not about becoming famous or rich, but rather about achieving something of real and enduring value that was aimed at revealing something fundamental about ourselves and our relationships with others as well as nature. The towering achievements of, for example, Michelangelo, Raphael, Rembrandt, and Titian, revealing humanity’s deepest emotions and relationships, are certainly difficult to compare with, for example, Claes Oldenburg’s displays of gigantic toilet seats and kitchen sinks, or Damien Hirst’s preserved carcasses.

In preparation for the celebration early next month of the centenary of the award of the Nobel Prize in Physics to Niels Bohr, arranged by the Royal Danish Academy of Sciences and Letters of which I am a member, I have been wondering whether the worries about lack of real ambition by contemporary artists might also be relevant to science. The Nobel Laureate Paul Nurse recently recollected a warning from Sydney Brenner, expressed when he received his Nobel Prize, about biologists “drowning in a sea of data and starving for knowledge.”^1^ As Paul Nurse stated,^1^ “That admonishment, from one of the founders of molecular biology, who established the nematode worm *Caenorhabditis elegans* as a model organism, is even more relevant to biology today.” Data are of course essential, but so are ideas, theories, and context. Data that do not lead to a theory or model, and therefore cannot be used to make predictions that can be tested, do not represent useful knowledge.

Alan Hodgkin understood this point better than most. In his Annual Review Prize Lecture, delivered at the Physiological Society’s centenary meeting in Cambridge in 1976, which I was privileged to attend, he recalled that virtually all the electrophysiological data from squid axons that went into the famous five Hodgkin and Huxley papers published in *J Physiol* in 1952, were collected in just 1 mo in the summer of 1949. The next 2 yr were spent on analysis, model building, and formulation of equations that finally allowed the reconstruction of the nerve action potential, demonstrating that the theory could predict correctly its precise shape and time course.^2^ The famous 1952 papers^3^ did not only represent useful knowledge but, as stated by the Ukrainian physiologist Oleg Krishtal, “The Nobel prizewinning papers by Alan Hodgkin and Andrew Huxley,^3^ in which they described nerve impulses in terms of ionic conductances, remain unsurpassed examples of beauty in science.”^4^ Interestingly, Hodgkin was not only concerned with the elegance and simplicity of his theories and papers, but also with the aesthetics of the experiments and the equipment used. In his magnificent review lecture,^2^ Hodgkin stated that, “an elegant piece of apparatus or an elegant experiment meant one that could be built or carried out very cheaply.” This view is certainly not fashionable today, but resonates with me as I recall^5^ the rather primitive, and partly self-built, equipment, Yoshio Maruyama and I used in 1982 to record the first single channel currents in epithelial cells.^6^

In today’s science world, in which the only title respected by increasingly powerful University Administrators, is “Mr or Ms Grant Holder,” the value of a scientist seems essentially to be assessed according to the size of his/her grant income. In contrast, one has the impression that there is generally little concern amongst university leaders about gaining real new knowledge. Inevitably, the primary ambition of the majority of scientists is therefore to obtain as much grant money as possible, as this is seen to be the most secure route to advancement. There is certainly little incentive to perform experiments with the cheapest possible equipment, as this would limit grant income and, most worryingly, there is little time to develop theories and new models because increasing proportions of time have to be spent on writing, and rewriting, grant applications. The absurdity of the current system is highlighted by remarks heard with increasing frequency, such as “I don’t have time to write (or read, or review) papers because I am too busy preparing yet another grant application.”

To re-establish a focus on what really matters, namely to gain useable knowledge from data, it would seem a good idea to rethink our working and assessment culture and start by placing much more emphasis on theory and model building as well as context.

## Data Availability

There are no data in this editorial.
